# Whole Genome Sequencing Reveals Potential New Targets for Improving Nitrogen Uptake and Utilization in *Sorghum bicolor*

**DOI:** 10.3389/fpls.2016.01544

**Published:** 2016-10-25

**Authors:** Karen Massel, Bradley C. Campbell, Emma S. Mace, Shuaishuai Tai, Yongfu Tao, Belinda G. Worland, David R. Jordan, Jose R. Botella, Ian D. Godwin

**Affiliations:** ^1^School of Agriculture and Food Sciences, The University of QueenslandBrisbane, QLD, Australia; ^2^Department of Agriculture and FisheriesWarwick, QLD, Australia; ^3^BGI-ShenzhenShenzhen, China; ^4^Queensland Alliance for Agriculture and Food Innovation, The University of QueenslandWarwick, QLD, Australia

**Keywords:** nitrogen uptake and utilization, nitrogen use efficiency (NUE), sorghum (*Sorghum bicolor)*, domestication, selection

## Abstract

Nitrogen (N) fertilizers are a major agricultural input where more than 100 million tons are supplied annually. Cereals are particularly inefficient at soil N uptake, where the unrecovered nitrogen causes serious environmental damage. *Sorghum bicolor* (sorghum) is an important cereal crop, particularly in resource-poor semi-arid regions, and is known to have a high NUE in comparison to other major cereals under limited N conditions. This study provides the first assessment of genetic diversity and signatures of selection across 230 fully sequenced genes putatively involved in the uptake and utilization of N from a diverse panel of sorghum lines. This comprehensive analysis reveals an overall reduction in diversity as a result of domestication and a total of 128 genes displaying signatures of purifying selection, thereby revealing possible gene targets to improve NUE in sorghum and cereals alike. A number of key genes appear to have been involved in selective sweeps, reducing their sequence diversity. The ammonium transporter (AMT) genes generally had low allelic diversity, whereas a substantial number of nitrate/peptide transporter 1 (NRT1/PTR) genes had higher nucleotide diversity in domesticated germplasm. Interestingly, members of the distinct race *Guinea margaritiferum* contained a number of unique alleles, and along with the wild sorghum species, represent a rich resource of new variation for plant improvement of NUE in sorghum.

## Introduction

Quantitatively, Nitrogen (N) is the most important nutrient required for plant growth and development and is often supplied in the form of synthetic N fertilizers. Their intensive commercial use and generation from fossil fuels, means N fertilizers have a high economic volatility and are often the most expensive additive used in achieving high yielding crops. Application can lead to a variety of environmental problems such as leaching into the waterways contributing to soil and water contamination, as well as the formation of nitrous oxide which plays a role in long-lived greenhouse gas accumulation (Vitousek et al., 1997; Lewandowski and Schmidt, 2006). One-hundred million tons of N fertilizers are used worldwide each year, 50% of which is applied to the three major cereals (Ladha et al., 2016). Cereals are inherently inefficient at N uptake resulting in only ~40% of total N applied being harvested in the grain (Peoples et al., 1995; Raun and Johnson, 1999; Sylvester-Bradley and Kindred, 2009). By the year 2050 world population is projected to reach nine billion (Tester and Langridge, 2010), pressuring farmers and breeders to increase commercial crop yields to meet the demand. Population growth is most accentuated in developing countries, including Sub-Saharan Africa, where there are limited resources for agriculture and input-intensive crops are already displaying depleting yields (Cardinale, 2012).

Nitrogen Use Efficiency (NUE) is a broad term incorporating various traits that can be targeted for improvement, such as N uptake or utilization. In cereals, NUE ultimately refers to maximizing grain N yield per unit of soil N (Good et al., 2004). It is often categorized into two subclasses: N Recovery Efficiencies (NRE) referring to the crops' ability to recover N from soil and N Internal Efficiencies (NIE) which refers to the crops' ability to efficiently assimilate and remobilize N. Many phenotypic traits can be manipulated, such as root morphology, total biomass and tiller number to affect NUE (Garnett et al., 2009; Van Oosterom et al., 2010a,b; Olson et al., 2013), however the efficiency of cellular pathways involved in N uptake and utilization also play an important role. For example, elite maize varieties often display enhanced utilization of N; but nonetheless, N uptake has remained constant throughout domestication, suggesting conventional breeding programs have reached NRE capacity (Moose and Below, 2009).

Nitrogen is most commonly available in soils in the inorganic forms of ammonium and nitrate, although amino acids and proteins can also be taken up (Paungfoo-Lonhienne et al., 2008; Näsholm et al., 2009). Uptake of ammonium and nitrate is a tightly regulated system facilitated by both constitutive and inducible molecular transporters that mediate their translocation into the root cells (Krapp et al., 2014). AMT2 and NRT1/PTR are low-affinity transporters which typically function in the presence of high levels of either ammonium or nitrate respectively (Tsay et al., 1993; Sohlenkamp et al., 2000; Neuhäuser et al., 2007; Yuan et al., 2007; Wang et al., 2012). Conversely, AMT1 and NRT2, with assistance from the accessory protein NRT3, function as high-affinity transporters which are most efficient under low concentrations (Masclaux-Daubresse et al., 2010; Zhou et al., 2015). NRT1/PTRs are a multigene family including several solute transporters with affinities for glucosinolates, di- and tri-peptides, as well as plant hormones (Léran et al., 2014; von Wittgenstein et al., 2014; Chiba et al., 2015).

Nitrate is converted into ammonium through enzymatic reactions carried out by Nitrate Reductase (NR) and Nitrite Reductase (NiR). Assimilation of ammonium depends on Glutamine Synthetase (GS), the gateway enzyme responsible for converting inorganic N into the organic amino acid glutamine, utilizing ATP. GS has two isoforms with distinct physiological functions. In higher plants, GS2 is typically encoded within a single gene where it functions in primary N assimilation within plastids. Alternatively, GS1 is encoded by a large multigene family where cytosolic GS1 is predominantly involved in ammonium recycling (Bernard and Habash, 2009). Glutamine is an important signaling molecule providing a negative feedback loop to reduce nitrate uptake through down-regulation of transporter genes as well as driving post-translational modifications (Miller et al., 2007). Glutamine is converted into two glutamate molecules by the enzyme Glutamine Oxoglutarate Aminotransferase (GOGAT) with the reducing power varies depending on isoform: NADH for cytosolic, or ferredoxin (Fd) for plastidic (Suzuki and Knaff, 2005). Within the mitochondria, Glutamate Dehydrogenase (GDH) balances relative levels of ammonium and glutamate (Suzuki and Knaff, 2005; Purnell and Botella, 2007).

Ultimately, glutamate is incorporated into aspartate and asparagine by reactions catalyzed by Aspartate Aminotransferase (AST) and Asparagine Synthetase (AS). Asparagine has the highest N:C ratio, making it a useful vehicle for long-range N transport and storage. These reactions are reversible, where Asparaginase (ASPG) converts asparagine to aspartate and Aspartate Ammonia Lyase (AAL) which converts aspartate to fumarate, each step releasing an ammonium ion. The four major amino acids, glutamine, glutamate, aspartate, and asparagine are typically the most abundant amino acids in plant roots and leaves (Lam et al., 1995; Paynel et al., 2001) where they are transported to sink tissues to fulfill their essential roles in various biosynthetic pathways (Forde and Lea, 2007; Pratelli and Pilot, 2014; Gaufichon et al., 2015).

Attempts to improve cereal NUE using breeding programs and genetic modification have mostly been unsuccessful due to the complexity of regulatory controls and a lack of accurate phenotyping (Garnett et al., 2015). Improvement of uptake efficiencies are difficult to achieve since uptake is dependent upon plant demand and is regulated through downstream assimilates where the mechanisms of regulation are poorly understood in cereals (Malagoli et al., 2005; Garnett et al., 2009, 2013). Overexpression of key enzymes such as NR, NiR, GS, and GOGAT has led to inconsistent results that often do not facilitate an improvement of NUE or phenotypic change (Yamaya et al., 2002; Good et al., 2004; Hirel et al., 2007; Masclaux-Daubresse et al., 2010). However, one example in maize involves two characterized GS1 genes involved in kernel number and size (Martin et al., 2006; Cañas et al., 2010) where constitutive overexpression of ZmGln1;3 increased kernel number (Martin et al., 2006). However, this was not reliably reproduced under field conditions (Thomsen et al., 2014). Constitutive expression of GDH from *Escherichia coli* and *Aspergillus* in tobacco and rice respectively resulted in biomass and seed size increases (Ameziane et al., 2000; Abiko et al., 2010). However, when two GDH genes from *Nicotiana plumbaginifolia* were expressed in tobacco, overall biomass was decreased (Terce-Laforgue et al., 2013). These inconsistent results demonstrate the lack of understanding of the intricate functions and regulation of key enzymes and their isoforms involved in the N assimilatory pathways.

Sorghum is an ideal candidate to study traits relating to NUE due to its higher grain yield per unit of N available from the soil during N limiting conditions, especially when compared to other major cereal crops (Lemaire et al., 1996; Hirel et al., 2007; Oikeh et al., 2007; Zhu et al., 2010; Vogan and Sage, 2011). Sorghum is also an important staple food for 500 million people in Asia and sub-Saharan Africa (Anglani, 1998). Its ability to tolerate periods of drought, high temperatures, and low inputs has made it a preferred cereal to grow in semi-arid climates. Domestication of sorghum occurred ~8000 years ago in the Eastern regions of Africa (Wendorf et al., 1992) and has given rise to the landraces examined within this study. Molecular evidence suggests a secondary domestication event occurred in Western Africa, where *Guinea margaritiferum* races originated (Mace et al., 2013). These sorghum accessions are morphologically distinct and cultivated in nutrient deficient soils (Mace et al., 2013; Morris et al., 2013). Studying population genetics throughout domestication and cultivation of sorghum will facilitate a clearer understanding of key genes facilitating NUE, as well as identifying genes maintaining allelic variation within a population, possibly as an adaption to variable soil environments.

Numerous traits have been selected for throughout domestication and improvement, the most obvious being grain number and size (Campbell et al., 2016), and lack of seed shattering. Other traits selected for throughout domestication and improvement include rounded seeds, photoperiod insensitivity, disease resistance, and reduction of seed tannins, however, depending on the end-use of the grains these features will vary throughout accessions. Landrace and wild and weedy sorghums have a larger overall biomass, mainly attributable to their height. Although assumed to require high available soil N, it is suggested that elite breeding lines have enhanced NIE as they typically remobilize 70–90% of plant N to the grain (Olson et al., 2013), and can continue to uptake significant levels of N post-anthesis (Sinclair et al., 1997; Kamoshita et al., 1998, 1999; Borrell and Hammer, 2000; Van Oosterom et al., 2010b). Thus, there is an expectation that key transporter genes involved in N storage and remobilization are more likely to be under strong selection within improved accessions, while genes involved in primary N uptake from the soil, especially under limited conditions, are more likely to be under selection in wild and weedy accessions.

This study provides the first broad population genetic analysis of the overall trends in the selection of genes involved in N uptake and utilization pathways in sorghum as viewed through the prism of domestication. Here we have identified key candidate genes for further targeted research with the ultimate aim of breeding cereal crops with improved NUE.

## Materials and methods

### Whole genome sequencing (WGS) and plant material

Resequencing data was previously described in Mace et al. (2013) wherein a total of 44 *Sorghum bicolor* genotypes underwent WGS by Illumina paired-end sequencing with ~22x coverage per line (16x–45x). The sequencing had a SNP calling accuracy of 99.85 and 99.72%, validated through targeted sequencing and whole-genome *de novo* assembly of representative lines. The accessions included are genetically representative of 18 Landrace and seven Wild and Weedy sorghums along with two *G. margaritiferums* and two progenitors *S. propinquum* genotypes (Table S1). Additionally, this analysis includes 19 inbred lines which are the product of various degrees of selective breeding.

### Identification of genes involved in N uptake and utilization

**Figure 1:**
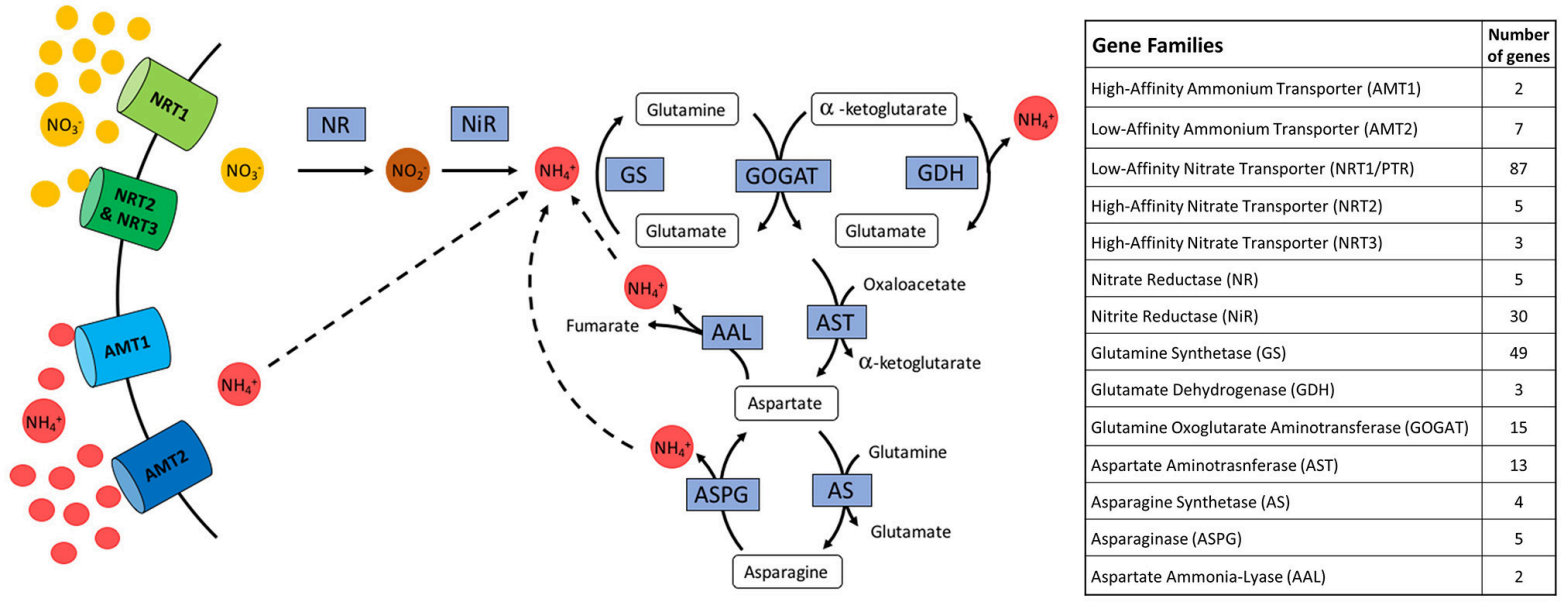
**Diagram of biosynthetic pathway in cereals of N uptake and utilization to generate glutamine, glutamate, aspartate, and asparagine shown with a table displaying relative number of genes for each identified transporter or enzyme**.

Nitrogen uptake commences with the recovery of nitrate and ammonium ions from the soil, followed by the rapid assimilation of these inorganic ions into organic forms (Figure 1). For the purposes of this study, the end point of the metabolic pathway was set at the generation and cycling of aspartate and asparagine, which facilitates the incorporation of N into four major amino acids: glutamine, glutamate, asparagine, and aspartate. Genes were grouped based on their gene family, where analysis was focused on the five transporter protein families, NRT1/PTR, NRT2, NRT3, AMT1, and AMT2; genes involved in N assimilation by two reductases NR and NiR, and conversions into glutamine and glutamate by GS, GOGAT, and GDH; and also within the aspartate and asparagine cycling involving AST, AS, ASPG, and AAL.

Identification of enzymes involved in N uptake and utilization were retrieved from the SorghumCyc (Tello-Ruiz et al., 2016) and Kyoto Encyclopedia of Genes and Genomes (KEGG) online sorghum pathway database (Kanehisa et al., 2012). Ammonium and nitrate uptake and transporter genes described by von Wittgenstein et al. (2014) and Léran et al. (2014) were added for analysis. All gene candidates were then filtered for expression utilizing the publicly available Morokoshi sorghum transcriptome database (Makita et al., 2015). Only those genes found to be expressed were used for analysis in this study, irrespective of expression levels. Of note, this database also includes expression data from sorghum exposed to N-stress, highlighting upregulation of genes involved in N stress response, however this analysis was limited to roots (Gelli et al., 2014). A total of 230 expressed genes were identified for potential involvement in the N biosynthetic pathway. Orthologous genes under selection identified via WGS studies of maize (Hufford et al., 2012; Jiao et al., 2012) and rice (He et al., 2011; Huang et al., 2012; Xu et al., 2011) were also compared to selected sorghum genes (Table S2). Genes within NRT1/PTR family were identified based on their homology with characterized Arabidopsis NTR1/PTR genes, as well as the unified nomenclature which divides these transporters into subfamilies and is denoted with a NPF number (Table S3; Léran et al., 2014; von Wittgenstein et al., 2014).

### Selection assessments

Population genetic assessments were performed on all identified genes to establish the effects of domestication and improvement upon genetic diversity, as well as determining if any genes displayed signatures of selection. To probe the direction and severity of selection, coding regions were analyzed using K_a_:K_s_ ratios which were computed using the software package Calculator 1.2 (MYN Method) following the protocol by Zhang et al. (2006). The K_a_:K_s_ ratio is the frequency of non-synonymous substitutions per non-synonymous site (K_a_) to the frequency of synonymous substitutions per synonymous site (K_s_). The ratio differentiates between positive selection if results are greater than one, purifying if less than one, and neutral if equal to one.

Analysis was performed at both whole gene and coding sequence (CDS) level for all 230 genes by evaluating nucleotide diversity (θπ; Nei, 1987), Watterson's estimator (θω; Watterson, 1975), and neutrality test *Tajima's D* (TajD; Tajima, 1989) using BioPerl modules with an in-house script (Mace et al., 2013). Further, F_ST_-values were calculated using an alternative BioPerl module with the same genetic components to estimate population differentiation. Signatures of purifying and balancing selection were identified utilizing the population genetic data mirroring the criteria outlined in Mace et al. (2013) and Campbell et al. (2016). Specifically, the thresholds applied to identify signatures of purifying selection were as follows; when θπ- and θω-values for a gene were in the lower 5% of the empirical distribution in the descendent population, F_ST_-values > 95% of the empirical distribution, and with a negative TajD. In contrast, the thresholds applied to identify signatures of balancing selection were as follows; when θπ- and θω-values were in the upper 25% of the empirical distribution in the descendent population, TajD in the upper 5% of the empirical distribution, and F_ST_ > 10% but lower than <90% of the population pair wise distribution.

Validation of genes under selection was performed using the mlHKA test (Wright and Charlesworth, 2004). Specifically, genes identified as being under purifying and balancing selection throughout domestication were compared against 34 neutral sorghum genes. The mlHKA test was run under a neutral model, the selection model was performed where numselectedloci = 0 and numselectedloci > 0. Mean log likelihood ratio test statistics were used to determine significance following a χ^2^ distribution where df equals the number of different parameters.

Genes were also analyzed for selection at the base pair level using the R package PopGenome (Pfeifer et al., 2014). Pairwise comparisons between ancestor/descendant populations were used to determine selection. To identify specific base pairs under purifying selection, the criteria utilized a reduction in diversity between pairwise population comparisons greater for the specific base pair than the mean gene diversity in the whole genome, an F_ST_ > 0, and a negative TajD-value. To identify balancing selection, the criteria included an overall increase in diversity between pairwise population comparisons, greater for the specific base pair than the mean gene diversity in the whole genome, F_ST_-values greater than zero, and a positive TajD-value.

### Haplotype network analysis of genes under selection

Haplotype network analysis was performed using the package PEGAS (Population and Evolutionary Genetics Analysis System; Paradis, 2010) in R and integrated using the ape (Paradis et al., 2004) and adegenet packages (Jombart, 2008) for genes under selection. In addition to the sorghum accessions that underwent population genetic assessments, with some exclusions, accessions from the *G. margaritiferum* race and *S. propinquum* were used in haplotype network analyses (Table S1), totaling 45 sorghum genotypes. This analysis was performed to validate selection and to establish how domestication events have shaped these haplotypes, with a particular focus on *G. margaritiferum* accessions which have resulted from a secondary domestication event (Mace et al., 2013). Haplotype network analysis were performed at both gene and CDS levels.

## Results

### N uptake and utilization pathway and sequence diversity

A combination of online databases and gene identification based on homology identified a total of 290 putative genes. Of these only those with published expression data were further analyzed (Makita et al., 2015). This resulted in a total of 230 genes potentially involved in the N uptake and utilization pathways (Figure 1). This pathway begins with uptake of either ammonium or nitrate through the transporter genes AMT1 (2 genes), AMT2 (7 genes), NRT1/PTR (87 genes; Table S3), NRT2 (5 genes), and NRT3 (3 genes). Nitrate requires assimilation into ammonium through the enzymatic roles of NR (5 genes) and NiR (30 genes), ammonium can then be converted into organic amino acid glutamine by GS (49 genes), and further into glutamate by GOGAT (15 genes) where GDH (3 genes) balances levels of glutamate and ammonium. Glutamate can be incorporated into the aspartate-asparagine shuttle which involves AST (13 genes), AS (4 genes), ASPG (5 genes), and AAL (2 genes). A subset of these enzymes require additional cofactors in order to function, such as the reducing agents ferredoxin (Fd) or NADH, as well as chemical energy from ATP.

**Figure 2:**
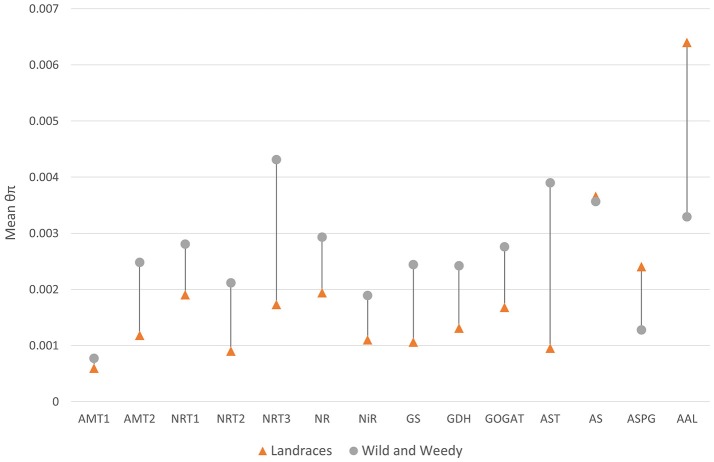
**The mean nucleotide diversity (θπ) for each gene family in Landrace and Wild and Weedy genotypes**. Analysis is performed on the CDS of each gene and averaged within each gene family within the nitrogen uptake and utilization pathway.

Of the 230 transcribed genes identified as putatively playing a role in N uptake and utilization, 79.5% (183 genes) displayed an average decrease in nucleotide diversity after domestication (Table S4). The examination of average nucleotide diversity within gene families revealed that there were reductions in sequence diversity in all the gene families in the early steps of the N pathway, up to the stage where glutamate is generated. Conversely, three gene families in the aspartate-asparagine cycling, specifically AS, ASPG, and AAL, displayed an increase in diversity (Figure 2). This disparity may be caused by the small number of genes within the families in the later stages of the pathway resulting in outliers having sizeable effects on the averages. Of the five ASPG genes, one gene (*Sobic.006G169500*) had a maximum of 32.7-fold increase of diversity (θπ_Landrace_ = 0.007699, θπ_Wild&Weedy_ = 0.0002358) in Landraces compared to the Wild and Weedy genotypes.

Amongst the 230 genes studied, eight genes displayed invariance at the nucleotide level throughout domestication (Table S5). Of these, AMT2 (*Sobic.003G400300*), a low affinity ammonium transporter gene, was invariant within all 44 sorghum accessions analyzed. Of the invariant genes, two were also determined to have significantly high F_ST_-values (F_ST_ > 0.5): a NiR gene (*Sobic.001G422300*) with F_ST:Domestication_ = 0.695 and an AST gene (*Sobic.002G413100*) with F_ST:Domestication_ = 0.593. Both also displayed signatures of purifying selection. The most predominant gene families displaying invariance were NRT1/PTR with three genes and AST with two genes.

### Signatures of selection

The K_a_:K_s_ ratio is a tool used to determine the direction and magnitude of selection. The majority of genes in all families showed a trend toward purifying selection with a K_a_:K_s_ ratio of less than one (Figure S1). GS genes had the highest K_a_:K_s_ ratio, while the ammonium transporters (AMT1 and AMT2) were low in all the sorghum genotype groups. Of the genes with a K_a_:K_s_ ratio less than one, 56 out of 146 genes in Landrace, and 77 of the 169 in Wild and Weedy groups were statistically significant (*p* < 0.05; data not shown). Average K_a_:K_s_ ratios throughout the N uptake and utilization pathway were K_a_:K_s:Landrace_ = 0.245 and K_a_:K_s:Wild&Weedy_ = 0.253.

Sixteen genes were found to display signatures of positive selection by having K_a_:K_s_-values greater than one in at least one genotype grouping and/or when comparing these groupings through domestication (Table 1). The gene families enriched with the high K_a_:K_s_ ratio were NRT1/PTR (9 genes), GS (5) genes, and NIR (2) genes.

**Table 1:** **List of genes that display pressures of positive selection within either Landrace or Wild and Weedy genotypes, and/or when comparing them throughout domestication**.

**Gene ID**	**Function**	**K_a_:K_s_ ratio**			**Nucleotide Diversity (θπ)**	
		**Landraces**	**Wild & Weedy**	**Domestication**	**Landraces**	**Wild & Weedy**
*Sobic.001G111100*	NRT1/PTR	1.574990	1.977870	NA	0.002041	0.003005
*Sobic.001G276300*	NRT1/PTR	0.336859	0.262895	0.49050	0.006815	0.006517
*Sobic.001G276600*	NRT1/PTR	0.226060	0.205998	1.39857	0.003121	0.004348
*Sobic.003G372000*	NRT1/PTR	NA	0.779389	1.91974	0.001054	0.003528
*Sobic.005G107500*	NRT1/PTR	1.740940	NA	2.43478	0.000925	0.001128
*Sobic.006G239000*	NRT1/PTR	0.333274	0.221838	1.33638	0.000407	0.001106
*Sobic.006G251200*	NRT1/PTR	1.120390	NA	1.25673	0.000636	0.000281
*Sobic.009G099000*	NRT1/PTR	0.000000	1.123060	0.56059	0.000828	0.001677
*Sobic.010G133100*	NRT1/PTR	0.252617	0.411819	1.73304	0.006464	0.006129
*Sobic.004G314200*	NiR	1.139690	0.517056	0.90156	0.000470	0.001648
*Sobic.008G006900*	NiR	NA	0.653504	1.35632	0.000667	0.001840
*Sobic.001G002000*	GS	3.256560	0.953878	1.05790	0.000615	0.000575
*Sobic.001G497300*	GS	1.011360	0.673694	NA	0.001000	0.001031
*Sobic.002G133400*	GS	0	1.712950	NA	0.000690	0.000802
*Sobic.006G210900*	GS	1.110000	0.211033	0.27145	0.001053	0.003297
*Sobic.007G078300*	GS	2.487300	3.282810	NA	0.000582	0.002025

K_a_:K_s_-values are given along with their respective nucleotide diversities, and those highlighted in gray display positive selection (K_a_:K_s_ >1).

Three genes were identified with signatures of purifying selection during domestication based on their sequence diversity (θπ), allele frequencies (TajD), and population differentiation (F_ST_; Table 2). All three of these genes were located in regions of the genome with selective sweeps, where adjacent genes were also determined to have signatures of purifying selection (Table 1). The three genes are NiR (*Sobic.001G42230*), AST *(Sobic.002G413100*), and GS (*Sobic.007G078500*), two of which (NiR and AST) are invariant throughout Landrace genotypes. All three genes were also shown to have high F_ST_-values: the NiR gene (*Sobic.001G422300*) with values of F_ST:Domestication_ = 0.695, the AST gene (*Sobic.002G413100*) with F_ST:Domestication_ = 0.593, and the GS genes *Sobic.007G078500* with F_ST:Domestication_ = 0.644.

**Table 2:** **List of genes and their population genetics data of those found to be displaying pressures of selection at the gene level throughout domestication**.

**Selection at gene level**	**Gene ID**	**Function**	**Selective Sweep (kb)**	**Nucleotide diversity (θπ)**		***Tajima's D* (TajD)**		**F_ST_**
				**Landraces**	**Wild & Weedy**	**Landraces**	**Wild & Weedy**	**Domestication**
Purifying	*Sobic.001G422300*	NiR	174.4	0	0.000764	NA	−0.345900	0.694667
Purifying	*Sobic.002G413100*	AST	188.0	0	0.013148	NA	1.273849	0.593150
Purifying	*Sobic.007G078500*	GS	79.4	0.000026	0.007312	−0.567739	1.464598	0.644061
Balancing	*Sobic.006G169500*	ASPG	–	0.007699	0.000236	2.001733	−1.313478	0.274193
Balancing	*Sobic.009G225700*	GOGAT	–	0.003671	0.002523	3.008451	−1.247962	0.188190

Two genes within the N uptake and utilization pathway exhibited evidence of balancing selection throughout domestication (Table 2). The ASPG gene (*Sobic.006G169500*) was identified with a signature of balancing selection throughout domestication with a substantial increase in nucleotide diversity of over 32-fold between the Landrace and Wild and Weedy genotypes (θπ_Landrace_ − 0.007699, θπ_Wild&Weedy_ − 0.000255). Both genes, the ASPG (*Sobic.006G169500*) and GOGAT (*Sobic.009G225700*) showed contrasting *Tajima's D*-values: the GOGAT with TajD_Landrace_ = 3.008 and TajD_Wild&Weedy_ = −1.248, and the ASPG gene with TajD_Landrace_ = 2.002 TajD_Wild&Weedy_ = −1.313.

Genes under selection throughout either domestication, or during subsequent breeding were confirmed using the mlHKA test of the genes under selection (Table S6). The N uptake and utilization pathway was found to display significantly fewer genes with signatures of purifying selection during domestication than expected (*p* = 0.0281), and also significantly fewer genes with signatures of balancing selection during improvement than expected (*p* = 0.0371). Chi-squared tests were also performed on the individual population genetics metrics for the genes in the N uptake and utilization pathways in comparison to the whole genome analysis (Table S7). Nitrate uptake transporter genes (NRT1/PTR, NRT2, and NRT3) within the Wild and Weedy genotypes had significantly fewer genes within the 5% tail of the empirical distribution of TajD-values in comparison to the whole genome.

Higher resolution analysis of CDS sequence at the base pair level of sorghum accessions identified 135 genes with signatures of purifying selection and 108 genes with signatures of balancing selection. Further examination revealed the majority are candidate domestication genes, where 128 genes presented signatures of purifying selection and 97 presented signatures of balancing selection (Tables S8, S9). The number of codons identified with signatures of purifying selection ranged from 1 to 21 in a single gene, with 14 genes containing at least 10 codons under selection. Two genes from the NRT1/PTR family contained the highest number of codons with signatures of selection: *Sobic.001G444000* (NPF8.11) with 21 codons under selection, of which nine were non-synonymous; and *Sobic.001G443900* (NPF8.7) with 16 codons under selection, two of which were non-synonymous.

The two genes with the highest number of codons under balancing selection were both from the GOGAT family: *Sobic.003G258800* with 23 codons, seven of which were non-synonymous; and *Sobic.009G225700* with 20 codons with signatures of balancing selection, of which 13 were non-synonymous. Both genes were determined to be displaying signatures of balancing selection at the gene level and also had codons with signatures of purifying selection. The first gene was GOGAT gene *Sobic.009G225700*, with seven codons with signatures of purifying selection, of which four were non-synonymous (three nonpolar and one acidic residue). Additionally, the ASPG gene (*Sobic.006G169500*) had one non-synonymous codon (nonpolar residue) with a signature of purifying selection.

### Haplotype network analyses

**Figure 3:**
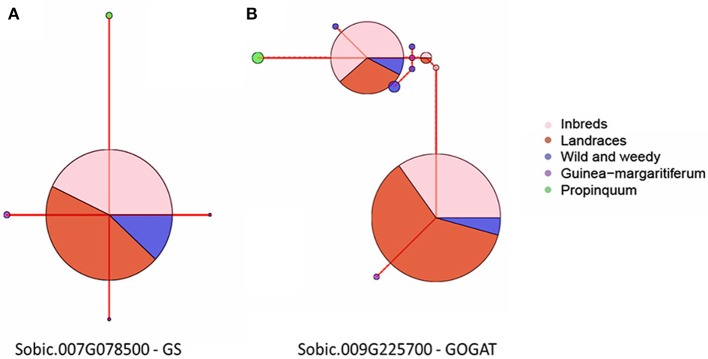
**Haplotype network analyses of the CDS of two genes found to be display signatures of selection during domestication at the gene level grouped into their sorghum genotype groups; Inbred Lines, Landraces, Wild, and Weedy, *Guinea margaritiferum, S. propinquum***. The size of the circle signifies the number of accessions within each haplotype and the red line joining the circles represents the relative variation between the haplotypes. **(A)** *Sobic.007G078500*, a GS gene under purifying selection. **(B)** *Sobic.009G225700*, a GOGAT gene under balancing selection.

**Figure 4:**
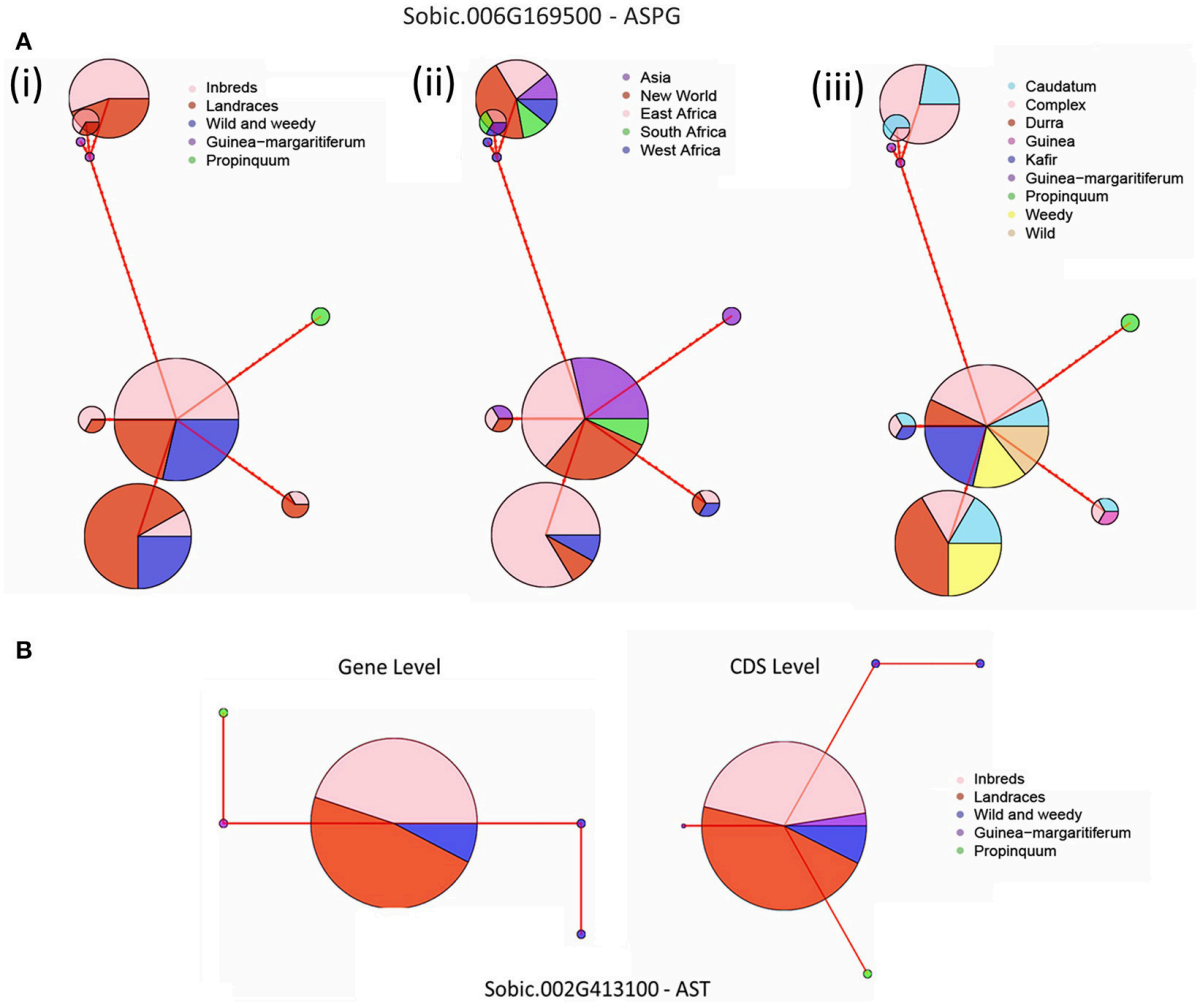
**Haplotype network analyses of genes under selection with suggestive results, where the size of circle signifies the number of accessions within each haplotype and the red line joining the circles represents the relative variation between the haplotypes**. **(A)** Gene level analysis of the ASPG gene Sobic.006G169500. The accessions are grouped according to: (i) genotype groups; Inbred Lines, Landraces, Wild & Weedy or race of *Guinea margaratiferum* and *S. propinquum*. (ii) geographic location; East Africa, West Africa, South Africa, Asia, New World and (iii) Racial grouping or species; caudatum, complex, durra, guinea, kafir, *Guinea margaritiferum, S. propinquum*, weedy, wild. **(B)** Gene and CDS level analyses of *Sobic.002G413100*, an AST gene that shares a major allele with *Guinea margaritiferum* grouped into their genotype groups.

Haplotype network analyses were performed on all five genes with signatures of selection identified at the whole gene level throughout domestication. This analysis was based on their genotype grouping (*S. propinquum, G. margaritiferum*, Wild and Weedy, Landrace and Inbred Lines), their geographical location (East Africa, West Africa, South Africa, Asia, New World) and race/species/wild (caudatum, complex, durra, guinea, kafir, *G. margaritiferum)*. Figures 3A,B show the common trends for two genes under purifying and balancing selection and illustrate the effect of domestication on these traits by either limiting diversity with one major haplotype or maintaining two or more prevalent haplotypes within cultivated genotype groups. The haplotype network analysis for the GS gene (*Sobic.007G078500*) identified with a signature of purifying selection at both whole gene and base-pair levels is presented in Figure 3A. Figure 3B shows the results of the haplotype network analysis for the GOGAT gene (*Sobic.009G225700*) under balancing selection at both gene and base-pair level throughout domestication. A contrasting example of a gene under balancing selection during domestication, *Sobic.006G169500* in the ASPG gene family is presented in Figure 4A, which maintains three high frequency haplotypes, one of which is dominated by the caudatum and complex racial types. Figure 4B displays the haplotype network analysis of the AST gene *Sobic.002G413100* which was found to share its haplotype with *G. margaritiferum.*

## Discussion

### Diversity within gene families

This study presents the largest evaluation of sequence diversity in genes within the N uptake and utilization pathway in a diverse panel of sorghum accessions and has identified a significant reduction of diversity in 80% of these genes as a result of domestication. This may be due to selective constraints on specific genes or blocks of genes, or the effects of an evolutionary bottleneck. The overall average reduction in diversity in genes within this pathway after domestication was in line with genome-wide averages (approx. 1.7-fold; Mace et al., 2013) and in the primary starch biosynthesis pathway (Campbell et al., 2016). These results uncover substantial amounts of unexploited genetic diversity within Wild and Weedy lines that could be evaluated by breeding programs. However, this trend was not observed for all gene families; AS, ASPG, and AAL displayed higher sequence diversity within the Landrace genotypes, although this may be attributed to the low number of genes within these families where outliers would exert substantial effects. The AAL gene family (2 genes) displays the highest nucleotide diversity, while the AMT1 family (2 genes) displays the lowest nucleotide diversity, representing differing pressures on gene families throughout the N uptake and utilization pathway.

NRT2, AMT1, and AMT2 gene family members had low nucleotide diversity in the Landrace grouping, suggesting selective constraint on all ammonium transporters and high-affinity nitrate transporters. The high-affinity nitrate transporter gene families (NRT2 and NRT3) co-function as a tetramer containing two subunits of each (Yong et al., 2010) and play a critical role in nitrate uptake as they are upregulated in roots during limited N conditions in sorghum (Gelli et al., 2014). An ancient whole-genome duplication in sorghum (Paterson et al., 2004) resulted in the formation of sub-functional protein complexes, and there are examples of asymmetrical evolutionary rates between such subunits (e.g., ADP-glucose pyrophosphorylase; Georgelis et al., 2008; Corbi et al., 2011). Similarly, the co-functioning gene families, which may have arisen from the duplication event, have contrasting nucleotide diversities with the NRT3 family having a 2-fold higher nucleotide diversity in both Landrace and Wild and Weedy genotypes. Four of the five NRT2 genes display SNPs with signatures of purifying selection, whereas the NRT3 gene family lacks any evidence of selection suggesting greater plasticity in NRT3 proteins. As NRT3 proteins induce initiation of lateral roots under low N conditions and modulate their growth under nutritional cues (Orsel et al., 2006), hypothetically, allelic variation within NRT3 genes may be beneficial for optimal transport of nitrate and root growth under variable environmental and soil conditions often found in subsistence farming systems.

Within the Landrace group, 20% of genes exhibited higher levels of nucleotide diversity, which is a preliminary indication of balancing selection, principally found within the NRT1/PTR gene family. Increases in nucleotide diversity can be attributed to a relaxation of selective constraint, or a desired increase to generate allelic variants that may be beneficial under subsistence agriculture N conditions. Surprisingly, the Inbred breeding lines, albeit a small subset of varieties with arguably similar genotypes to Landrace, still demonstrate an increase of diversity in 45% of genes within the N uptake and utilization pathway. These genotypes includes sorghums generated as sweet sorghum types, as well as those developed for a variety of farming practices in Australia, USA, China, India, and countries in west and east Africa. Thus, the variability in desired breeding outcomes may have resulted in this allelic diversity. An example of this is two NRT1/PTR genes, *Sobic.001G444000* & *Sobic.001G444100*, both within the NPF8 family which includes functional peptide transporters (PTR) with no affinity for nitrate (Komarova et al., 2008; Weichert et al., 2012; Léran et al., 2014). Both these genes are invariant within the Landrace category as well as the majority of the additional breeding lines analyzed. The exceptions are within two sorghum genotypes Rio and E-tian, both of which are sweet sorghums. Surprisingly, this variant allele is also similar to those of the two *Guinea-margaritiferums*. This finding provides some evidence to suggest that varying end uses may have placed different selection pressures on sorghum's nitrogen uptake and utilization pathway.

Six other genes are invariant within Landrace accessions. One belongs to the NRT1/PTR family, where *Sobic.003G304000* is within the NPF1 subfamily and functions as a high-affinity nitrate transporter in Arabidopsis (Léran et al., 2014), suggesting this gene is also a critical transporter protein within sorghum. Additionally, an AMT2 gene *Sobic.003G400300*, is invariant among all 44 sorghum lines analyzed. Based on preliminary expression profiles, this gene is likely involved in the mobilization and recycling of ammonium during early grain filling (Makita et al., 2015). As this gene does not have a homologous gene in rice or maize, it may represent a species-specific adaptation within N uptake and utilization in sorghum. This strict selective constraint is likely due to a combination of its crucial function and inflexibility of protein sequence.

### Purifying selection

Signatures of purifying selection were identified in three genes at the whole gene level during domestication: a NiR, an AST and a GS gene. These are likely optimally functioning isoforms and may contribute to phenotypes improving agronomic performance, and therefore are worth further characterization in sorghum. Two of these genes, the NiR and AST were also invariant throughout Landrace genotypes. However, all of these genes were located within a broader selective sweep where surrounding genes also displayed similar pressures of selection and/or invariance. Since the majority of sorghum genes are uncharacterized it is difficult to determine the drivers of such selective pressures.

Additionally, this pathway contains less than expected number of genes displaying purifying selection in comparison to the sorghum genome as a whole. This may be attributable to the lack of methods available to phenotype NUE during crop domestication and thus, genes involved in N uptake and utilization were only indirectly selected for. The N uptake and utilization pathway also requires high levels of flexibility, where Landrace sorghums are placed on soils with a range of fertility, climate conditions, end uses and differing farming practices adopted by an assortment of ethnically and culturally diverse people. Thus, combined genomic analyses of such diverse sorghums may result in the loss of signals of purifying selection, whereas analyzing sorghum subpopulations taken from similar soil types and geographical locations may allow for the discovery of additional genes under purifying selection.

### Balancing selection

Genes identified with signatures of balancing selection maintained several alleles in the population conferring a selective heterozygous advantage and likely function optimally under varied conditions according to specificities in development, tissue, or stressors. Two genes were determined to be under balancing selection at the whole gene level throughout domestication. Although only one GOGAT gene family member was identified with signatures of balancing selection at the gene level, two were identified with at least 20 codons displaying pressures of balancing selection throughout domestication (*Sobic.009G225700* and *Sobic.003G258800*). GOGAT isoforms can have distinct physiological functions (Vance et al., 1994; Lam et al., 1995), where various genes display cell-specific and organ-specific patterns of expression with differential regulation (Edwards et al., 1990; Thum et al., 2003; Suzuki and Knaff, 2005), and as such, the GOGAT genes may play important roles under variable N conditions.

An ASPG gene (*Sobic.006G169500*) was identified with a signature of balancing selection at both the whole gene level and base-pair level with 11 codons under balancing selection (four non-synonymous—two polar, one nonpolar, one basic) while also having the largest increase in nucleotide diversity throughout domestication. With the above exception, haplotype network analyses of genes displaying pressures of balancing selection generally indicate that heterozygosity is independent of genotype grouping, race, and geographical location. Modern hybrids are generated from crossing two inbred parental lines with genotypes from different racial origins, typically a caudatum-derived line and either a kafir- or durra-derived line. Caudatums are highly utilized in breeding as they typically have the largest and roundest seeds, resulting in higher yields and a reduction in net loss during post-harvest processing (Doggett, 1988; Dahlberg, 2000). It is possible the dominant use of caudatums in breeding has forced pressures of balancing selection upon certain loci based on racial structure, as the haplotype network analysis demonstrates one of the core alleles is dominated by caudatum and complex races. Such diversity within other genes displaying balancing selection cannot be attributed to racial structure, and is more likely the result of adapting to varied environmental conditions throughout domestication. Further characterization is required to confirm if heterozygous plants confer a selective advantage.

### Positive selection

Positive selection is the accumulation of relatively high levels of amino acid altering mutations, modifying the function of the gene. Signatures of positive selection were determined in 22 genes, most frequently within NRT1/PTR and GS gene families, with a few examples in NiR. In cases such as the NRT1/PTR gene *Sobic.001G111100* (NPF8.5) and the GS gene *Sobic.007G078300*, values demonstrate positive selection in both Landrace and Wild and Weedy sorghums. The former, *Sobic.001G111100* has been described within the NPF8 subfamily involved in the transport of dipeptides (Léran et al., 2014), but may have an altered function in sorghum. Another gene of interest belonging to the GS gene family (*Sobic.001G002000*), came under strong positive selection in Landrace genotypes as well as during domestication. As the GS family likely contains multiple key isoforms, this gene is of particular interest as it may be adapted specifically for subsistence agriculture practices and would be worth exploring variants of this gene in Landrace genotypes.

Two genes *Sobic.009G099000* (NRT1/PTR, NPF2.1) and *Sobic.002G133400* (GS) underwent positive selection exclusively in Wild and Weedy lines. This suggests these genes may be adapting to limited or variable N supply, making them ideal candidates to gain insights into potential modes for improving NUE under low N conditions. The NRT1/PTR gene (*Sobic.009G099000*—NPF2.1) is within the subfamily of nitrate transporters involved in both the efflux of nitrate in mature roots and known as a nitrate excretion transporter (NAXT), critical during early embryo development (Segonzac et al., 2007; Almagro et al., 2008). Alternatively, this NAXT gene in Wild and Weedy may be under exclusive positive selection as Wild and Weedy sorghums are perennials in comparison to cultivated sorghums which are semi-perennials grown as annuals. Thus, changing the root system to operate for longer periods of time, potentially reducing the investment into N assimilation systems. Further characterization of genes displaying such selection could shed insights into sorghum's adaptability to low and varied N conditions within its Wild and Weedy and Landrace groups.

### Orthologous NUE improvement genes

Nitrogen uptake and utilization genes in sorghum were compared to homologous genes in maize and rice to determine if similar pressures of selection have been identified in the closely related cereals. Orthologs of 22 maize and six rice genes were found to have signatures of selection throughout their domestication (He et al., 2011; Huang et al., 2012; Hufford et al., 2012; Jiao et al., 2012). However, of these, only three orthologous genes in sorghum (*Sobic.003G295500*—NRT1/PTR, *Sobic.007G125900*—GS, *Sobic.006G169500*—ASPG) were identified with similar selection pressures at the gene level. Additionally, a GS gene (*Sobic.005G213200*) displayed evidence of selection in rice, maize, and sorghum. Comparative analysis studies on pressures of selection and SNP analysis may be informative for other crops since sorghum is a good model for more complex genomes, such as maize and sugarcane.

The gene family member NRT1.1B (NPF6.5) was identified with signatures of selection in both sorghum and rice. In rice, it has been demonstrated that a variant allele contributes to increased nitrate absorption (Hu et al., 2015). The NPF6 subfamily of NRT1/PTR transporters have been found to be involved in dual-affinity nitrate transport (Léran et al., 2014). Additionally, two rice genes described as short panicle 1 (*Os02g48570*—NPF7.11 & *Os11g12740*—NPF4.1) play roles in tillering and panicle elongation (Li et al., 2009; Wang and Li, 2011) and are also found under selection in maize (Hufford et al., 2012; Jiao et al., 2012). The homologs in sorghum are both NRT1/PTR genes (*Sobic.004G260500*—NRT1.5, NPF7.9 & *Sobic.005G090600*—NPF4.1) and each display a synonymous SNP under selection during domestication. They are within differing subfamilies, both of which in Arabidopsis are not always involved in transport of nitrate (Léran et al., 2014), however, *AtNRT1.5* has been linked to xylem loading of nitrate (Lin et al., 2008). Therefore, exploring variations of this gene within diverse sorghums for varying efficiencies of nitrate uptake and transport is of interest.

In maize, the GS1 gene *ZmGln1;3* improves kernel number and size when constitutively overexpressed (Martin et al., 2006) and was identified as being under selection, and shares 96% protein identity with the sorghum gene *Sobic.004G24700*, however, the sorghum ortholog lacks evidence of selection. In rice, overexpression of *GS1;1* (*Os02g50240*) and *GS1;2* (*Os03g0223400*) improved soluble N content within the seeds (Cai et al., 2009; Thomsen et al., 2014). *GS1;1* shares 87% protein identity, and *GS1;2* gene shares 95% protein identity with *Sobic.004G247000*, the same GS1 protein sharing high identity within the maize studies. However, overexpression of this gene in maize transformation studies has led to inconsistent results whereby previous results cannot be reproduced or have not been validated in the field (Martin et al., 2006; Cañas et al., 2010; Thomsen et al., 2014). The limited data available on overexpression, even in one of the most studied enzymes within this pathway, highlights the gaps in our knowledge and the need for further research.

### Candidate genes for NUE improvement within sorghum

Re-introduction of diversity within genes can be accomplished by utilizing the diversity from Wild and Weedy accessions, cultivated *G. margaritiferums* or the Asian species *S. propinquum*. Utilizing exotic germplasm could be beneficial for a variety of improvements, as diversity is often viewed as the key to adaption. This is especially true within the N uptake and utilization pathway as available N sources are often highly variable, even within modern agriculture systems. High affinity transporter genes (NRT2/NRT3, AMT1) often have low nucleotide diversity in cultivated accessions in comparison to Wild and Weedy accessions and are therefore important candidates for the re-introduction of diversity. In rice, *OsNRT2.3* has two splice variants, where *OsNRT2.3B* is expressed in the phloem and regulates nitrate transport activity through sensing pH levels (Cai et al., 2008; Feng et al., 2011). High activity of this transporter enhances the uptake of N, iron, and phosphate through enhancing the pH buffering capacity of the plant, where field trials resulted in a 40% increase in NUE (Fan et al., 2016). The homologous gene in sorghum is *Sobic.003G270800*, an NRT2 gene that displays an increasing nucleotide diversity throughout domestication and improvement, potentially demonstrating selection in modern breeding for increased diversity. For other sorghum NRT2 genes which are not displaying such an increase, it would be worth exploring variants within Wild and Weedy populations. These high-affinity transporter genes are ideal NUE targets and should be introduced into cultivated material to assess their value in modern production systems.

Other candidate genes for NUE include the three genes found under purifying selection at gene level. The corresponding alleles for these genes within the *G. margaritiferum* genotypes were often quite varied and only demonstrated shared haplotypes in two cases. The first is NRT1/PTR gene *Sobic.003G185100* (NPF6.2) which has homologs in maize (*GRMZM2G064091*) and rice (*Os01g37590*). In Arabidopsis, this gene is described as *AtNRT1.4* and is most highly expressed within the petiole, which is linked to storage of nitrate under high N supply (Chiu et al., 2004; De Angeli et al., 2006; Tsay et al., 2007). If this gene retains a similar function in sorghum, it could represent a key gene for nitrate storage or remobilization in leaf sheaths, the grass equivalent to the dicotyledonous petiole. The second gene is an AST gene (*Sobic.002G413100*) that displays pressures of purifying selection during domestication and is invariant throughout Landrace accessions analyzed. Interestingly, this gene only shares the haplotype with *G. margaritiferum* races of sorghum in the CDS, whereas at the gene level its haplotype is not as closely related. This likely occurred through accumulation of synonymous mutations where it has 13 SNPs displaying pressures of purifying selection during domestication, six of which were synonymous. It is also possible this represents differential regulation of this gene between the two domestication events, where the dissimilarity at the gene level is within regulatory or non-CDS regions, and would be worth characterizing. Thus, typically the *G. margaritiferum* genotypes present high levels of genetic diversity which could be utilized for improving NUE.

## Conclusion

This study focused on the analysis of genes within the N uptake and utilization pathway and has successfully identified a number of key gene targets for further research. Due to limited experimental data regarding sorghum's N assimilation pathway, the vast majority of these genes are uncharacterized and function could only be assumed based on homology. The genes discussed herein may not be an exhaustive list and ultimately may not function as suggested within this study. For example, the NRT1/PTR gene family encompasses a variety of transporters involved in the transport of proteins and other compounds, and not all members will be linked to the uptake and transport of nitrate. Additional bioinformatics analyses concerning diverse sorghums grown under known soil types and fertilizer regimes would be beneficial for breeding programs to produce lines adapted to specific geographic locations and farming practices. Furthermore, performing gene-based association mapping within NAM populations is also worth exploring to link genes and their allelic diversity to NUE phenotypes.

A more complete understanding of the components involved in this pathway is vital toward the progress of improving NUE. Specifically, transcriptome data of sorghum grown under varied environmental conditions, especially in low N levels, could elucidate key stress-induced genes and their respective promoters. Previous overexpression studies often used constitutive promoters, but tissue-specific or inducible promoters have been suggested to yield more consistent results, especially under limited N conditions, and may avoid negative feedback loops that have hindered results of previous studies (Thomsen et al., 2014; Garnett et al., 2015). Due to the tight regulation evident within this pathway, genetic modification is likely required to introduce variants or alter proteins in order to increase the efficiency of enzymes under specific conditions. This would avoid turning off upstream and downstream reactions, while focusing on modifying key SNPs within genes that display pressures of selection. It is with optimism that further characterization of key genes and elucidation of regulatory networks involved in N cycling of plants will facilitate the development of NUE crops.

## Author contributions

Conception and design of the work was substantially performed by KM, BC, IG, JB, and DJ. Majority of the sequence data was generously given by EM and DJ and statistically analyzed by KM, BC, ST, YT, and additional analysis into transporter genes was performed by KM and BW. The manuscript underwent revision and was given final approval by all.

## Funding

This work was funded by the Grains Research and Development Corporation (GRDC) project UQ76 and Australian Research Council (ARC) projects DP0986043 and DP140102505 and LP0990626, as well as The University of Queensland, School of Agriculture and Food Sciences–Queensland.

### Conflict of interest statement

The authors declare that the research was conducted in the absence of any commercial or financial relationships that could be construed as a potential conflict of interest. The reviewer SPR and handling Editor declared their shared affiliation, and the handling Editor states that the process nevertheless met the standards of a fair and objective review.

## Supplementary material

Figure S1 **A comparison of the mean K_a_:K_s_ ratios for each gene family within the N uptake and utilization pathway between Landrace (orange triangle) and Wild & Weedy (gray circle) genotype groups**.

Table S1 **Description of sorghum accessions used in the population genetics study, their category, country of origin, general geographic location, and if they were included in the haplotype network analyses**. This includes, 18 Landraces, seven Wild and Weedy, 19 Inbred Lines, two *Guinea margaritiferum*, and two *S. propinquum* accessions.

Table S2 **The 230 genes analyzed within the N uptake and utilization pathway displaying orthologous maize and rice genes**.

Table S3 **List of NRT1/PTR genes with their respective NRT1/PTR supergroups, homology to Arabidopsis, and NPF family**.

Table S4 **The 230 identified genes putatively involved in N uptake and utilization in sorghum with their population genetic analyses (**θπ**, TajD, F_ST_)**. These are organized into their gene families and in the order of the biosynthetic pathway. Genes with a reduction in diversity throughout domestication and improvement have a white background in the nucleotide diversity section, while those with an increase in diversity during only domestication are green, throughout only improvement are gray, and throughout both domestication and improvement are blue. Genes with high F_ST_-values (>0.5) are highlighted in yellow.

Table S5 **List of invariant genes during domestication and their respective population genetic data**. Highlighted in green is *Sobic.003G400300* which is invariant throughout all sorghum lines analyzed. High F_ST_–values (>0.5) are highlighted in yellow.

Table S6 **Statistical Tests (A) mlHKA test results of 3 genes under purifying selection and 2 under balancing selection compared to 34 neutral genes**. Seven runs were performed with *p*-values validating selection. **(B)** The 34 neutral genes used as input and their chromosome and bp start and stop site.

Table S7 **χ^2^ analysis on the frequency of sorghum population genetic analyses within 5 and 95% tails grouped according to gene families**. Significant results are highlighted in yellow. Only significant results were shown.

Table S8 **List of 128 genes that display codons under purifying selection during domestication**. Includes base pair position, codon, amino acid, and whether a polymorphism is synonymous or non-synonymous.

Table S9 **List of 91 genes that display codons under balancing selection during domestication**. Includes base pair position, codon, amino acid, and whether a polymorphism is synonymous or non-synonymous.
