# Supplementary figures and images for: Whole Genome Sequencing Reveals Potential New Targets for Improving Nitrogen Uptake and Utilization in Sorghum bicolor
Source: Front Plant Sci. 2016 Oct 25;7:1544. doi: 10.3389/fpls.2016.01544 (PMC5078838; doi:10.3389/fpls.2016.01544)

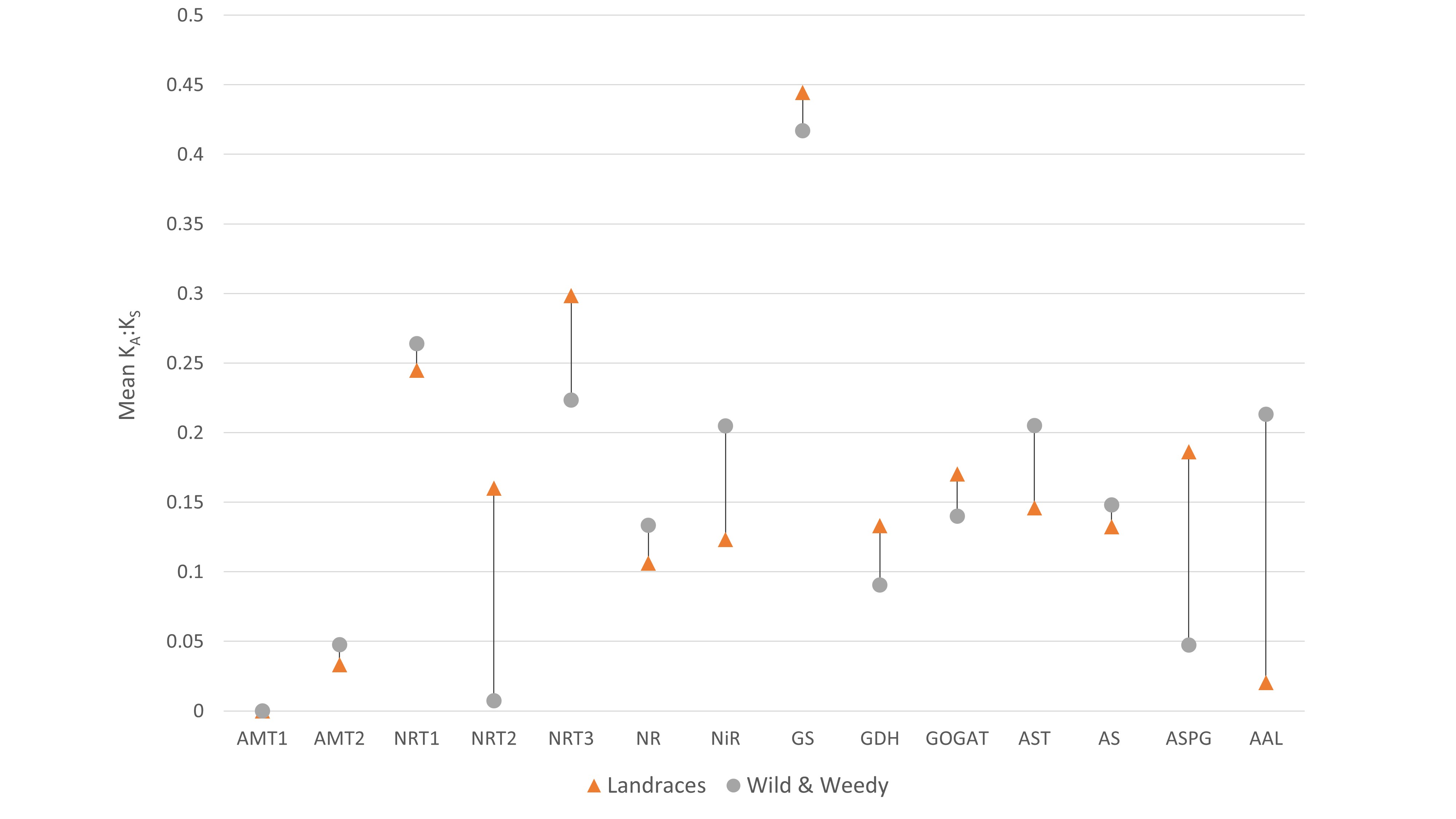

Supplement: Figure S1 — A comparison of the mean Ka:Ks ratios for each gene family within the N uptake and utilization pathway between Landrace (orange triangle) and Wild & Weedy (gray circle) genotype groups. [file Image1.JPEG]
